# Domestic feline contribution in the transmission of *Sporothrix* in Rio de Janeiro State, Brazil: a comparison between infected and non-infected populations

**DOI:** 10.1186/s12917-018-1340-4

**Published:** 2018-01-18

**Authors:** Pãmella A. Macêdo-Sales, Simone R. L. S. Souto, Carolina A. Destefani, Ricardo P. Lucena, Ricardo Luiz D. Machado, Marcia R. Pinto, Anderson M. Rodrigues, Leila M. Lopes-Bezerra, Elisabeth M. S. Rocha, Andréa Regina S. Baptista

**Affiliations:** 1 Applied Microbiology e Parasitology Postgraduation Program, Fluminense Federal University (UFF), Niterói, RJ Brazil; 2 Veterinary Medicine Postgraduation Program, Fluminense Federal University (UFF), Niterói, RJ Brazil; 3 Medical and Molecular Mycology Laboratory, Microbiology and Parasitology Department, Biomedical Institute, Fluminense Federal University (UFF), Rua Prof. Hernani Melo, 101 São Domingos, Niterói, RJ CEP: 24210-130 Brazil; 4 Microbiology, Immunology and Parasitology Department, São Paulo Federal University (UNIFESP), São Paulo, SP Brazil; 5 Cellular Mycology and Fungal Proteomics Laboratory, Rio de Janeiro State University (UERJ), Rio de Janeiro, RJ Brazil

**Keywords:** Sporotrichosis, Subcutaneous infection, Zoonosis, Cat (*Felis catus*), Neglected diseases

## Abstract

**Background:**

Sporotrichosis is a neglected zoonosis caused by pathogenic fungi belonging to the *Sporothrix schenckii* complex. In Rio de Janeiro state, this disease reached an epidemic status with over 4700 domestic felines and around 4000 humans affected since the mid-90s. The present study evaluated clinical and epidemiological aspects and also the frequency of colonization and infection by these fungi in healthy cats and among those with suspicious cutaneous lesions, inhabiting four Rio de Janeiro state distinct areas.

**Results:**

Three hundred and seventy-one cats were included in two groups: 175 healthy cats [CRG] and 196 cats showing lesions suggesting sporotrichosis [SSG]. Mycological diagnosis allowed SSG animals to be divided in positive [104 cats; +SG] and negative [92 cats; -SG] groups. Nails, oral mucosa and lesions swabs were submitted to culture and potential colonies were subculture for micromorphologycal analysis, dimorphism and molecular tests. In the CRG, only one cat was colonized in the oral cavity [0.57%]; in the -SG group, four animals showed colonization of the nail and/or oral cavity [4.3%]; while the highest frequency of colonization [39.4%] was observed in the +SG. All molecularly typed isolates were identified as *S. brasiliensis*.

**Conclusion:**

The results obtained here indicate that healthy cats have a minor role in sporotrichosis transmission within the state of Rio de Janeiro. Conversely, a higher participation of diseased feline in sporotrichosis transmission was evidenced, especially by the colonization of their oral cavity. *Sporothrix brasiliensis* equally affects and colonizes animals from distinct Rio de Janeiro state areas. Thus, we hypothesize that sporotrichosis is a uniform endemic throughout the state, whose transmission depends mainly on the contact with cats with sporotrichosis. Since Rio de Janeiro displays a world unique epidemic model of the disease, not fully understood, data on the infected and non-infected animals can be of major importance for future strategies of sporotrichosis prevention and control. Finally, considering the importance of the current concept of “one health”, the experience here observed can be helpful for distinct epizootias and/or zoonosis.

## Background

Sporotrichosis is a subcutaneous mycosis caused by pathogenic dimorphic fungi belonging to the recently described *Sporothrix schenckii* complex, associated with plant organic matter and/or decaying matter in hot and humid climate regions and usually [1–3]. The disease was formerly known as Rose gardener’s disease because of its acquisition through traumatic inoculation of plant organic matter, but it is currently considered a zoonosis because of its transmittance through scratches or bites of cats. In fact, the feline particular behavior exposes them to possible environmental niches of the fungi in its saprophytic phase since scratching tree trunks and other surfaces, burying their feces and licking their bodies, facilitates nails and nasopharynx/oral cavity to carry particles of these fungi [4, 5]. Therefore, these animals are at higher risk for *Sporothrix* spp. conidia colonization and subsequent infection, playing a key role in the transmission of this zoonosis because they promote the traumatic implantation of the fungi in the subcutaneous tissue of humans and of other animals and also because they have an exuberant clinical presentation of sporotrichosis with intense proliferation of the parasitic form of the fungus [4–6].

In feline sporotrichosis, the spectrum of lesions ranges from self-limiting forms with the presence of a single lesion that may regress spontaneously to fatal systemic clinical cases. The difficulties regarding the treatment of feline sporotrichosis, such as prolonged treatment time, high cost, drug dosage forms and isolation of affected animals, often contribute to their abandonment, which eventually die [7–12]. Currently, soil contamination by infected carcasses represents a major environmental concern because it can greatly increase the spread of fungi and hold back the control of this mycosis, closing the cat-environment-man cycle [10, 13].

Sporotrichosis has been described in the last decades as one of the most important zoonosis and also as a neglected disease, having reached an epidemic status in the state of Rio de Janeiro [7, 9, 13] with over 4700 domestic felines [14] and around 4000 humans [13] affected since the mid-90s. Until 2009, approximately 3244 cats, 2200 humans and 120 dogs presenting this subcutaneous mycosis were reported by the most important sporotrichosis reference center in Rio de Janeiro, the National Institute of Infectious Diseases of the Oswaldo Cruz Foundation (IPEC-FIOCRUZ) [10]. Still, all these cases were reported from a single reference center/institution [14]. Therefore, there is a pressing need for larger investigations in order to provide a broader view of sporotrichosis in its different aspects such as epidemiology, fungal biology and environmental particularities of transmission within this state. Thus, it is clear that sporotrichosis has reached an epidemic magnitude in the only Brazilian state wherein notification is compulsory and the disease is considered an important zoonosis [8, 13, 15]. Additionally, although in reduced numbers compared to Rio de Janeiro, sporotrichosis zoonotic transmission by domestic felines have been reported in other Brazilian states [16]. Given its relevance, routine laboratory procedures for investigating its etiological agent are well established and include the in vitro isolation of *Sporothrix* spp., identification of these fungi based on their macro and microscopic features, and in vitro thermal conversion techniques. Nevertheless, the epidemiological data show that the disease still affects humans and domestic felines with growing frequencies, especially for this state.

Several authors have investigated the clinical-epidemiological, immunological and molecular aspects of sporotrichosis, and they have primarily focused on the metropolitan region of Rio de Janeiro [2, 4, 8, 11, 15, 17, 18]. Despite these studies, data on the presence of the fungi on the nails and in the oral cavity of cats that do not carry the disease as well as of those infected are inconclusive. Indeed, the few studies that have addressed this issue in Brazil have been restricted to the states of Rio Grande do Sul and São Paulo and also the metropolitan region of Rio de Janeiro [4–6, 9, 16, 19, 20]. None of these studies included domestic felines inhabiting areas other than the metropolitan region of the state of Rio de Janeiro.

The aim of the present study was to compare the potential role of *Sporothrix* infected and non-infected domestic feline in the transmission of sporotrichosis by investigating colonization and infection by these fungi in cat populations from different regions of Rio de Janeiro state, Brazil.

## Methods

The inclusion of animals was performed regardless of breed, age or gender, and the study was conducted over a period of 12 months (August 2012–2013). Similarly, the owners were informed of the objectives and methodology of the study and were asked to sign an informed consent form and answer questions related to epidemiological variables. Animals from the distinct location covered by the studied area were included. Likewise, cat populations from west, central-south and north regions of the municipality of Rio de Janeiro (Capital), Baixada Fluminense (Metropolitan region I, including Duque de Caxias, Nilópolis, São João do Meriti and Magé cities), Grande Niterói (Metropolitan region II – including Niterói, São Gonçalo and Itaboraí cities), Mountain (including Teresópolis and Petrópolis cities), Baixada Litorânea (Cabo Frio, Araruama, Arraial do Cabo, Búzios, São Pedro da Aldeia and Saquarema cities) and central-south Fluminense (Areal city) regions, as described by the Health State Secretary of Rio de Janeiro (SES/RJ), were investigated in the present study.

All animals were submitted to clinical evaluation by the veterinarians of this study, and clinical and epidemiological factors, such as gender, age, castration and contact with soil/plants and other domestic animals, were evaluated. All data were registered on a standardized data collection form. A total of 371 animals were included and divided into groups according to the presence/absence of skin lesions indicative of sporotrichosis as follows: 196 cats with clinical suspicion of sporotrichosis (Suspicious Sporotrichosis Group – SSG) and 175 apparently healthy cats (Colonization Research Group - CRG).

The following biological samples were collected based on clinical conditions, type of lesion and availability at the time of the study: swab from the lesion exudate; swab of the oral cavity; and nail tip fragments from both thoracic members. For diagnostic purposes, cytopathology analysis was conducted by impression smears of the skin lesion prepared on clean and dry glass slides, subsequently stained by the quick panoptic method (Laborclin, Pinhais, PR, Brazil), a Romanowsky-type stain. The slides were analyzed by light microscopy using 40× and 100× objective lenses for the identification of *Sporothrix* yeast-like suggestive structures. All the clinical specimens were immediately transported to the Medical and Molecular Mycology Laboratory, Biomedical Institute of the Fluminense Federal University (LMMI-UFF). Samples that were collected with a swab (lesion exudate and oral cavity) were submitted to routine mycological examination as follows: seeding onto Sabouraud agar 2% dextrose (BD, Franklin Lakes, NJ, USA) and Mycosel® (BD, Franklin Lakes, NJ, USA). The nail tip fragments were processed according to the method developed by the LMMI-UFF staff. Briefly, 5 × 7 mm plastic Ziploc bags containing 5–10 nail tips were used for storage, and the tips were subsequently transferred to 2.0 ml Eppendorf tubes containing 500 μl of 0.9% sterile saline solution. These tubes were submitted to vortexing for 15 s to dislodge possible adhered particles and then centrifuged at 1677 *xg* (Hettich Zentrifugen, Tuttlingen, Germany) for 15 min. Thirty microliters of the obtained pellet were then transferred and streaked on two 90 × 15 mm Petri dishes containing Sabouraud agar 2% dextrose (BD, Franklin Lakes, NJ, USA) and Mycosel® (BD, Franklin Lakes, NJ, USA). All media were incubated at room temperature (25–28 °C) and observed over four weeks for fungal growth. As previously described [3], colonies showing features suggestive of the *Sporothrix* genus were subcultivated on Sabouraud agar 2% dextrose (BD, Franklin Lakes, NJ, USA) at room temperature for colony isolation. The fungi were initially identified based on macro- and microscopic characteristics. Later, potential colonies were subcultured in brain–heart infusion agar (BHI; BD, Franklin Lakes, NJ, USA) at 37 °C for conversion to the yeast stage of *Sporothrix*. A random sampling subset on each study group/area, comprising approximately half of all studied isolates (*n* = 51), was submitted to molecular identification according to a species-specific PCR targeting the calmodulin gene (*CAL*), as previously described [21]. Briefly, fungal cells were recovered from 10-day-old colonies grown on Sabouraud dextrose agar, further used for genomic DNA extraction with the Fast DNA kit (MP Biomedicals, Vista, CA, USA), according to the manufacturer’s protocol. DNA extracts concentrations were estimated with a NanoDrop 2000 spectrophotometer (Thermo Fisher Scientific, USA) and stored at − 20 °C until use. We performed a 25 μL reaction as a final volume for the PCR experiments, conducted as follows: 12.5 μL PCR Master Mix buffer (2×), which consisted of 3 mM MgCl^2^, 400 mM each dNTPs, and 50 U/mL Taq Polymerase (Promega Corporation, Madison, WI, USA); 9.5 μL water, 1 μL each of forward and reverse primers (10 pmol/μL; Integrated DNA Technologies, USA), and 1 μL of target DNA [100 ng/μL], using the touchdown PCR method on a Eppendorf Mastercycler Pro machine (Eppendorf, Hamburg, Germany). The resulting PCR amplicons were separated and visualized on 1.2% agarose gel electrophoresis for 1 h at 100 V in the presence of GelRed (Biotium, Hayward, CA, USA). The L-Pix Touch (Loccus Biotecnologia, São Paulo, Brazil) imaging system was used to visualize the stained bands under UV light.

The data were processed and analyzed with the aid of statistical software BioEstat 5.3 (Belém, PA, Brazil). To analyze the results, the chi-square test and risk estimate test were used, and the nature of the variables was considered. For all tests, the level of significance was set at *p* < 0.05.

## Results

### Epidemiological and clinical evaluation

**Fig. 1:**
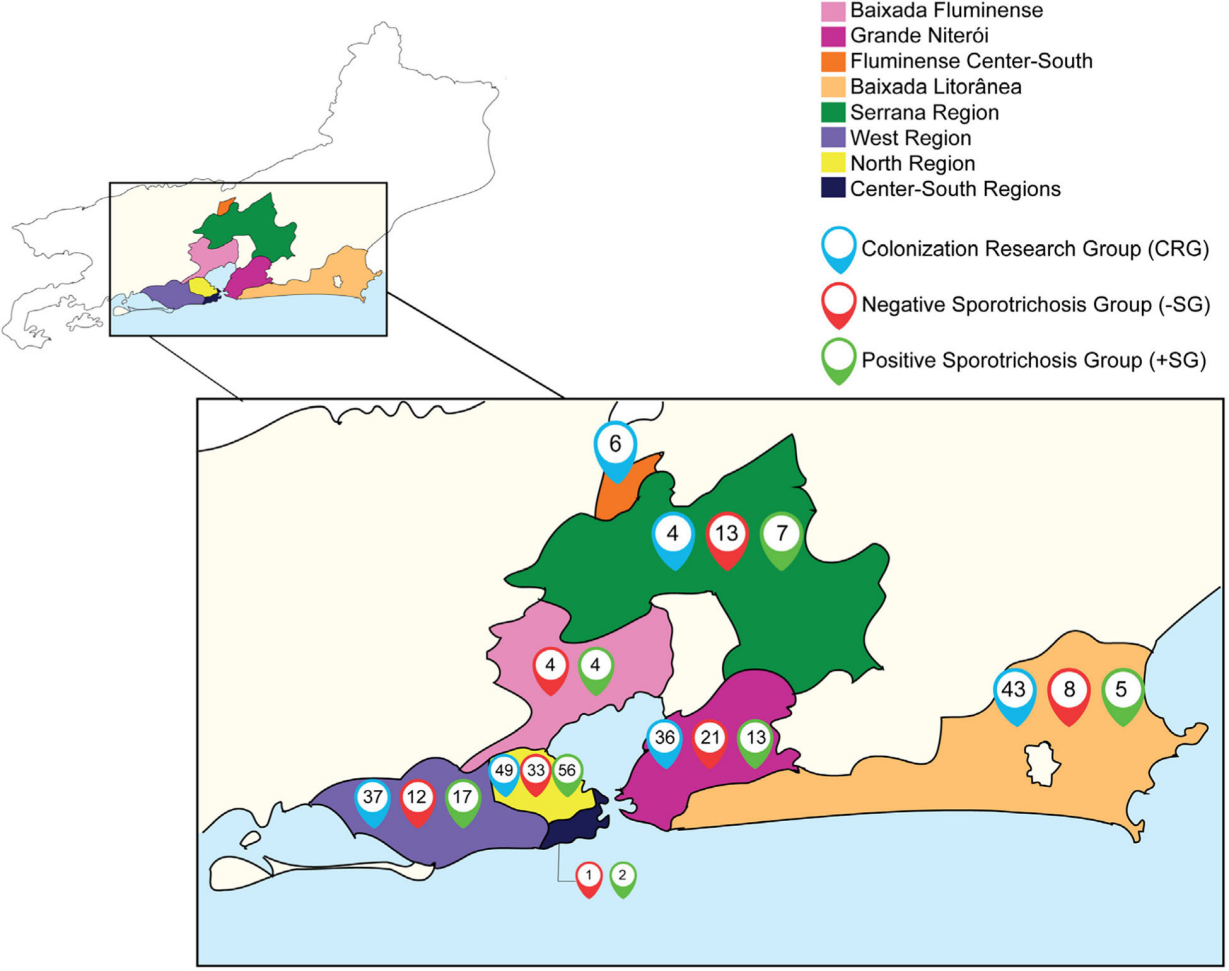
Map of the state of Rio de Janeiro highlighting the regions inhabited by the cats included in the present study. In each region, the absolute number of cats and groups to which they belong are depicted: CRG (Colonization Research Group), Negative Sporotrichosis Group (-SG) and Positive Sporotrichosis Group (+SG)

Of the 371 animals included in the present study, most inhabited the northern region of the capital (Metropolitan region I; 37%; 138/371), with the remaining cats distributed as follows: Grande Niterói (Metropolitan region II; 19%; 70/371), western region of the capital (Metropolitan region I; 18%; 66/371), Baixada Litorânea (15%; 56/371), Mountain region (6%; 24/371), Baixada Fluminense (Metropolitan region I; 2%; 8/371), Central-south Fluminense region (2%; 6/371) and Central-south capital region (1%; 3/371; Fig. 1). Table 1 shows the distribution of clinical-epidemiological data of the cat populations according to the study group, Colonization Research Group (CRG; *n* = 175) or Suspicious Sporotrichosis Group (SSG; *n* = 196), with the latter group divided into two subgroups after laboratory diagnosis by culture (gold-standard): +SG, Positive Sporotrichosis Group (*n* = 104); and -SG, Negative Sporotrichosis Group (*n* = 92; other skin pathologies). A comparison of cat populations of the different geographical areas showed they were similar in all investigated clinical-epidemiological parameters (*p* > 0.05).

**Table 1:** Absolute number and percentage (%) distribution of cats according to group and clinical-epidemiological variables

Study populations	CRG *n* (%)	SSG		TOTAL *n* (%)	*p*-value
Clinical-epidemiological variables		-SG *n* (%)	+SG *n* (%)		
	175	92	104	371	
Age (mean in years)	2.85 ± 2.8	4.45 ± 3.9	3.75 ± 3.1	3,51 ± 3,2	
Male	88 (50.3)	41 (44.6)	62 (59.6)	191 (51,5)	0,035^ϯ^
Neutering	100 (57.1)	52 (56.5)	47 (45.2)	199 (53,6)	0,037*
Undefined Breed (UDB)	129 (92.1)	82 (95.3)	92 (94.8)	303 (93,8)	
Urban Area	133 (76.0)	78 (84.8)	82 (78.8)	293 (79,0)	
Contact with Soil/Plants	145 (82.9)	68 (73.9)	75 (72.1)	288 (77,6)	0,034*
Contact with Animals	164 (93.7)	80 (87.0)	76 (73.1)	215 (57,9)	0,0004*
Contact with Cats with Sporotrichosis	59 (33.7)	24 (26.1)	40 (38.5)	123 (33,2)	
Risk Event Prior to Lesion	n.a.	28 (30.4)	43 (41.3)	71 (36,2)	

*CRG* Colonization Research Group, *SSG* Sporotrichosis Suspicion Group, *-SG* Negative Sporotrichosis Group, *+SG* Positive Sporotrichosis Group

*p < 0.05 between CRG and +SG; Chi-square Test

^ϯ^*p* < 0.05 between -SG and +SG; Chi-square Test

n.a.: not analyzed

The mean age of the studied domestic feline population was 42 months (σ ± 39 months), ranging from 2 months to 22 years. There was no significant difference in the mean age of the animals among the three groups studied, even when age was stratified in different groups (< 24 months, 24 to 60 months and > 60 months; *p* > 0.05; data not shown). Approximately half of the cats were male (51.5%), and 58.4% of the population was neutered. No differences were observed in the number of females and males between the +SG and CRG groups (*p* = 0.124). However, the +SG group had more males (59.6%) than the -SG group (44.6%; *p* = 0.035) and a higher number of unneutered animals (54.8%) compared with CRG (42.9%; *p* = 0.037).

A comparison of the cat breed, nutritional status, and area of origin (urban or rural) among the +SG, CRG and -SG groups did not present significant differences (*p* > 0.05). Most of the animals were identified as undefined breed (UDB; 93.8%; 303/323), some were identified as Persian (1.5%; 5/323) and Siamese (4.2%; 14/323), and only one was Himalayan (0.3%; 1/323). Most of the study population inhabited urban areas (CRG: 76%; -SG: 84.8%; +SG: 78.8%), and fight with other animals was the most often reported (36.2%) risk event prior to the suspicion of sporotrichosis lesion (SSG), followed by contact with other animals with sporotrichosis without any record of fight (25%). The majority (83%) of the CRG animals had contact with soil or plants in the house where they lived, and this potential exposure to the fungi was higher than in the +SG animals (72.1%; *p* = 0.034; Table 1). In the CRG, over half of the population was free-roaming (60%), and owners reported that the majority (93.7%) of cats shared their home environment with other animals. In turn, animals with sporotrichosis had less contact with other animals in their own houses (+SG: 73.1%; *p* = 0.0004) compared with the healthy group (CRG).

**Fig. 2:**
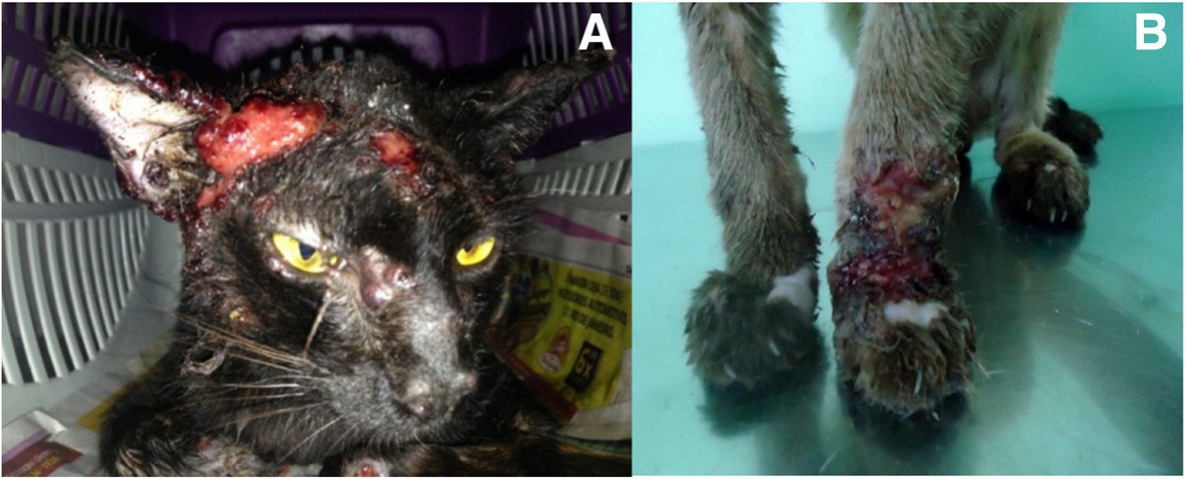
Most commonly observed sporotrichosis cutaneous lesions in two infected domestic feline showing crust, ulceration, blood and pus in the cephalic region (**a**) and thoracic member (**b**)

Crust, ulceration and blood and pus represented the most frequent macroscopic aspects of lesions in infected cats (Fig. 2; Table 2). In many animals, pruritus in varying degrees was reported (69.6%) as a clinical symptom concomitant with lesions; however, this clinical sign was not preferentially observed in cats with sporotrichosis (+SG versus -SG; *p* = 0.299; Table 2). Sneezing and weight loss were more commonly found among +SG cats (sneezing: 62.5%, 65/104; weight loss: 26%, 27/104; Table 2) when compared with -SG animals (sneezing: 39.1%, 36/92; weight loss: 13%, 12/92). Sneezing in cats with suspicious skin lesions was associated with a greater probability of positive sporotrichosis diagnosis (38.6%; *p* = 0.001; OR: 0.386; 95% CI: 0.217 to 0.687).

**Table 2:** Absolute number and percentage (%) of cats according to group and clinical variables

Populations	+SG *n* = 104 (%)	–SG *n* = 92 (%)	*p*-value	OR (IC95%)
Clinical Signs				
Respiratory Distress	37 (35.6)	24 (26.1)		
Nasal Secretion	51(49.0)	33(35.9)		
Sneezing	65(62.5)	36 (39.1)	0.001	0.386 (0.217–0.687)
Lack of Appetite	2 (1.9)	3 (3.3)		
Weight Loss	27 (26.0)	12 (13.0)	0.024	0.428 (0.202–0.904)
Apathy	20 (19.2)	18 (19.6)		
Ocular Secretion	3 (2.9)	2 (2.2)		
Epistaxis	5 (4.8)	1 (1.1)		
Macroscopic Aspect of Lesions				
Crust	61 (58.7)	41 (44.6)	0.049	0.567 (0.321–0.999)
Peeling	5 (4.8)	8 (8.7)		
Erythema	31 (29.8)	20 (21.7)		
Ulceration	77 (74.0)	45 (48.9)	< 0.001	0.336 (0.184–0.611)
Presence of Pus	31 (29.8)	13 (14.1)	0.009	0.388 (0.188–0.797)
Presence of Blood	71 (68.3)	39 (42.4)	< 0.001	0.342 (0.191–0.614)
Nodule	38 (36.5)	23 (25.0)		
Depigmentation	5 (4.8)	1 (1.1)		
Vesicle	7 (6.7)	2 (2.2)		
Degree of Pruritus				
Absent	29 (27.9)	26 (28.3)		
Mild	24 (23.1)	22 (23.9)		
Moderate	22 (21.2)	11 (12.0)		
Severe	24 (23.1)	23 (25.0)		

*+SG* Positive Sporotrichosis Group, *-SG* Negative Sporotrichosis Group

*OR* odds ratio

### Infection with pathogenic fungi of the *S. schenckii* complex

Mycological examinations revealed colonies that showed both macro- and micro-morphological characteristics of the *Sporothrix* genus*.* The macromorphological characteristics of the colonies included an initially cream brown color that turned to a dark brown color after 5–7 days of incubation and a dark brown color with velvety aspect and rugged topography at 25 °C. The colonies showed a cream color at 37 °C, and some colonies showed a creamy aspect and smooth surface, whereas others showed a friable aspect and rugged surface, all compatible with *Sporothrix* pathogenic fungi*.* The species-specific PCR showed that all 51 isolates correspond to *S. brasiliensis*: 25 from sporotrichosis lesions (+SG = northern region: 8; Grande Niterói: 4; western region: 5; Baixada Litorânea: 2; Mountain region: 3; Baixada Fluminense: 2; Central-south capital region: 1), 12 from nails (+SG = northern region: 4; Grande Niterói: 3; western region: 2; Baixada Litorânea: 2; Baixada Fluminense: 1), 13 from oral mucosa (+SG = northern region: 4; Grande Niterói: 4; western region: 2; Baixada Litorânea: 2; Baixada Fluminense: 1), plus one from the oral mucosa in the CRG group; all obtained from different domestic feline, as detailed below.

Swab samples from the exudate of lesions suggestive of sporotrichosis and at least one slide obtained via imprint were obtained from 196 animals (SSG), and 53% (104/196; +SG) showed a positive diagnosis, with no confirmed diagnosis in the remaining cats with suspicious skin lesions (-SG).

There was no difference in the frequency of infected animals among the surveyed geographic regions (χ^2^ = 3.878; *p* = 0.6932), distributed as follows: central-south region, north and west regions of the capital (67–59%), Baixada Fluminense (50%) and, finally, Grande Niterói, Baixada Litorânea and Mountain region (38–35%).

### Colonization versus studied groups

#### CRG (colonization) and –SG (Sporotrichosis negative diagnosis)

Table 3 summarizes the frequency of colonization by fungi of the *Sporothrix* genus in animals of the three groups according to anatomical site (nails and/or oral mucosa). *Sporothrix brasiliensis* was isolated by culture in a single case of the “healthy” group (CRG; 0.57%, 1/175) from an animal inhabiting the northern region of the municipality of Rio de Janeiro. As for the -SG animals, the same agent was isolated from four cats (4.3%) of the Baixada Fluminense, Mountain region, Grande Niterói and northern region, and one of these animals was colonized both in the nail and in the oral mucosa.

**Table 3:** Frequency of colonization by *Sporothrix* in the three groups according to anatomical site

	Nail Fragments	Oral Cavity	Both Sites	Total *n* = 371 (%)
CRG n = 175 (%)	0	1 (0.57%)	0	1 (057%)
-SG n = 92 (%)	1(1.1%)	2(2.1%)	1(1.1%)	4(4.3%)
+SG n = 104 (%)	11(10.6%)	12(11.5%)	18(17.3%)	41 (39.4%)

*CRG* Colonization Research Group, *+SG* Positive Sporotrichosis Group, *-SG* Negative Sporotrichosis Group

#### +SG (Sporotrichosis positive diagnosis)

The highest frequency of colonization was observed in the population with sporotrichosis at 39.4% (41/104; *p* < 0.0001). The northern region of the capital and Grande Niterói showed a similar frequency of colonized animals (47.6 and 50%, respectively). In these regions, cats with colonized nails represented 33.3 and 25% of the population, whereas those with colonized oral mucosa were 19 and 25%, respectively. Among the diseased and colonized animals living in the western region of the capital, half were colonized in the oral mucosa and the other half at both anatomical sites. The same percentage was observed in the population of Baixada Fluminense. In Baixada Litorânea, half of the cats with sporotrichosis carried the fungi in the nails, while the other half had this agent in the oral cavity.

### Nail fragments and oral mucosa colonization

A total of 340 nail fragments and 190 swabs of the oral cavity mucosa were obtained from the 371 evaluated cats (Table 4). Overall, 31 nail fragments were colonized (9.18%; 31/340), whereas 34 swabs of the oral mucosa (17.9%; 34/190) were positive.

**Table 4:** *Sporothrix* isolation within different biological samples

	CRG (p/n)	+SG (p/n)	-SG (p/n)	Total (p/n)
Nail Fragments	0/174	29/86*	2/80	31/340
Oral Cavity	1/66	30/66*	3/57	34/190

*CRG* Colonization Research Group, *+SG* Positive Sporotrichosis Group, *-SG* Negative Sporotrichosis Group; p = positive

*Differences were not observed in colonization between oral cavity and nail fragments (χ^2^ = 2.158; p = 0.1418)

In the CRG, the prevalence of fungal isolation in the oral cavity was 1.49% (1/66); however, none of the nail fragments were colonized (0/174). As for the -SG, the fungi were isolated from two nail fragments (2.5%; 2/80) and three swabs of the oral cavity (5.3%; 3/57).

The highest frequency of colonized clinical specimens was observed in animals with sporotrichosis (+SG) with 33% of nails (29/86) and 54% of the oral mucosa swabs (36/66) testing positive; significant differences were not observed between these sites (χ^2^ = 2.158; *p* = 0.1418).

## Discussion

Since the first report of sporotrichosis zoonotic transmission in 1998, the disease has shown a progressive increase in the number of human and animal cases, most of them admitted at the National Institute of Infectious Diseases of the Oswaldo Cruz Foundation (IPEC-FIOCRUZ), which is the reference center for diagnosis and treatment of the disease [2, 4, 7, 9, 17, 18, 20]. Thus, the available literature on clinical-epidemiological data of sporotrichosis in the state of Rio de Janeiro is limited to these populations. The present study investigated infection and colonization by pathogenic fungi of the *S. schenckii* complex in domestic feline populations with or without sporotrichosis from different regions of the state of Rio de Janeiro.

One of the postulated hypotheses was that specific clinical-epidemiological features of cat populations inhabiting different geographic regions would be observed because of the remarkable climatic and demographic differences between these regions, especially the Mountain region and Baixada Litorânea versus the metropolitan areas (Capital, Grande Niterói and Baixada Fluminense). Such a result would be expected because both the Mountain and Baixada Litorânea regions have milder climate and lower population density and are geographically distant from the Baixada Fluminense, which represents the initial epicenter the epidemic. However, the groups of domestic cats surveyed were similar in age, sex, breed, nutritional status, environmental exposure to the fungi, origin (rural or urban) and neutered status, regardless of the region studied. One possible explanation is the fact that both the Mountain region and Baixada Litorânea represent popular tourist regions, which increases the movement of people and subsequently favors the displacement of their animals to these regions [22].

The mean age of the different domestic feline populations of the +SG was homogeneous among the groups, as reported by Madrid et al. [5]. At approximately 3 years of age, cats have reached sexual maturity and come into heat, increasing the chance of fighting for females and subsequent exposure to infected animals. This hypothesis is corroborated by the fact that the studied cats with suspicious lesions (SSG) were involved in fights prior to the onset of the wounds. Despite the predominance of males, this difference was significant only when +SG was compared with the group with other skin pathologies (-SG). The higher number of females in the (-SG) group is noteworthy because females could have been affected by dermatophytosis or parasite infections such as scabies, which are acquired independent of trauma [23]. As expected, a higher number of unneutered animals was observed in the infected population, signaling an increased risk of exposure to the etiological agent of sporotrichosis because of fights over territory and females [4, 9, 15]. Interestingly, cats here defined as “healthy” (CRG) had greater contact with environmental niches compared to animals with sporotrichosis (+SG). Taken together, these results reinforce that feline sporotrichosis in Rio de Janeiro is acquired from other cats affected by this mycosis rather than by direct contact with environmental sources.

Cutaneous lesions in cats with sporotrichosis were compatible with previously described wounds in different cat populations including crust, ulceration and presence of pus and blood [4, 5, 20, 24], as well as respiratory signs and weight loss [20, 24] with the sneezing being the most frequent. Weight loss could be a result of respiratory distress, which may lead to eating difficulties. Data on clinical manifestations of feline sporotrichosis point toward a closer attention by veterinary medicine professionals to respiratory symptoms, especially sneezing, associated with weight loss, crusted lesions, and blood and pus as frequent symptoms of this disease.

Different authors reported the isolation of *Sporothrix* from environmental sources [1, 16, 25]. Surprisingly, nearly all of the 175 healthy cats inhabiting Rio de Janeiro endemic areas did not harbor the fungus either in their oral cavity or their nails, although the great majority of them had prior contact with soil or plants [1, 25, 26]. The single oral cavity colonized cat within this group lived in a cattery and shared the same feeder and drinker with other cats with diagnosed sporotrichosis. Therefore, we postulate that this animal’s colonization began by sharing such fomites.

In Table 5 we summarize published data on *Sporothrix* spp. colonization in different Brazilian domestic feline populations. The low frequency of colonization in the healthy cat population from distinct Rio de Janeiro state regions (0.57%) detected is consistent with those reported for cats living in its capital [4] (Table 5; 3.57%) and in the city of São Paulo [19] (SP; Southeast Brazil; 0.7%, 1/120). The authors attributed the low percentage of isolation to the fact that São Paulo is not an endemic area for sporotrichosis. In contrast, a study performed in Rio Grande [27] (RS; South Brazil) in a shelter with 90 animals with or without sporotrichosis, reported fungal isolation from the nails of seven healthy cats (29.1%) amongst all 24 healthy ones as detected by direct claw impressions in a culture medium. This finding may be associated with the presence of *Sporothrix* conidia in the paws or in soil fragments attached to them and not necessarily to the nail exclusive colonization. Considering these results, we conclude that environmental exposure may not be a determining factor for colonization in cats of the surveyed areas. Therefore, nails of non-infected cats are not important as a fungal inoculum reservoir, greatly diminishing the importance of trauma caused by healthy animals in this zoonosis transmission.

**Table 5:** Scientific articles on *Sporothrix* colonization in cats from different Brazilian regions, including the present study

Author/Year	Location	CRG (n)	+SG (n)	-SG (n)	Technique used to investigate the nails	Frequency of isolation in the nails (%)			Frequency of isolation in the oral cavity (%)			Frequency of isolation in the nasal cavity (%)		
						CRG	+SG	-SG	CRG	+SG	-SG	CRG	+SG	-SG
Schubach et al. [4]^c^	Rio de Janeiro/RJ	84	148	–	Seeding of the *pool* of nail fragments	–	39.5	–	3.57	41.8	–	–	66.2	–
Schubach et al. [15]^c^	Rio de Janeiro/RJ	10	347	–	–	–	–	–	–	49,1	–	0	70,5	–
Souza et al. [27]^d^	Rio Grande/RS	24	–	–	Paw impressions in culture medium	29.1	–	–	–	–	–	–	–	–
Leme et al., ^d^ [20]	Rio de Janeiro/RJ	12	11	–	–	–	–	–	16.7	45.5	–	25	72.7	–
Madrid et al. [5]^c^	Rio Grande do Sul/ Brasil^a^	–	92	–	–	–	–	–	–	45.2	–	–	–	–
Borges et al. [19]^d^	São Paulo/SP	119	1	–	Paw impressions in culture medium	0	0.7	–	–	–	–	–	–	–
Present study^c^	Rio de Janeiro/ Brasil^b^	175	104	92	Seeding of the pool of nail fragments	0	33	2.5	1.49	54	5.3	–	–	–

*CRG* Colonization Research Group, *+SG* Positive Sporotrichosis Group, *-SG* Negative Sporotrichosis Group

^a^Study performed in eight municipalities of Rio Grande do Sul (Rio Grande, Pelotas, Capão do Leão, Pedro Osório, Morro Redondo, Canguçu, São Lourenço and Bento Gonçalves)

^b^Animals in this study inhabited the following areas of the state of Rio de Janeiro: west, central-south and north regions of the city of Rio de Janeiro, Baixada Fluminense, Grande Niterói, Serrana and dos Lagos Regions and central-south Fluminense

^c^ Data referring to clinical specimens

^d^ Data referring to domestic cats surveyed

Four animals with suspicious of sporotrichosis and negative mycological culture (-SG) were colonized by *S. brasiliensis* in their nails and/or oral mucosa (Tables 3 and 5). These four animals lived with other cats diagnosed with sporotrichosis, except by one of them. Noteworthy, this last domestic feline had been under treatment with itraconazole for 23 months and was persistently colonized in the oral mucosa. Therefore, the present study stands out, for the first time, the potential for sporotrichosis transmission even by cats undergoing antifungal therapy for a long period and with negative mycological results.

Among cats with sporotrichosis here investigated, over half (54%) were colonized in the oral cavity, while approximately one-third were colonized in the nails (33%; Table 3). As summarized in Table 5, these results are similar to previous studies performed in cat populations from Rio de Janeiro capital [4, 15] as well as from Rio Grande do Sul [5]. As expected, the highest frequency of colonization occurred among domestic feline with sporotrichosis since the these animals have the habit of cleaning themselves by licking and also licking the wound itself where this yeast is abundant [4, 5]. In addition, as pruritus was repeatedly noted in the lesions of several animals here included (approximately 70%), it may have served as stimulus for increasing licking, thus favoring the maintenance of these fungi in the oral cavity. Nasal and oral cavities were more often colonized compared with the nails (present study, [4, 5, 20]), indicating that bites would be the traumatic event with the greatest potential for sporotrichosis transmission rather than scratching.

## Conclusions

Because of distinct urbanization, socioeconomic level, climate and demographic aspects of each surveyed area in the present study, which could affect fungal environmental distribution, differences in the sporotrichosis Rio de Janeiro endemics were expected. Nevertheless, in addition to the city of Rio de Janeiro and Baixada Fluminense region, *Sporothrix* equally affects and colonizes animals from other areas, including Grande Niterói, Baixada Litorânea and the Mountain region. Furthermore, our data confirm *S. brasiliensis* as the most prevalent species in the Brazilian feline sporotrichosis epidemics. Thus, we hypothesize that sporotrichosis is a uniform epidemic throughout the state, whose transmission depends mainly on the contact with cats with sporotrichosis.

Colonized feline populations with or without sporotrichosis are concentrated in Rio de Janeiro, São Paulo and Rio Grande do Sul states, and despite their marked geoclimatic differences uniform results are observed. The healthy cat population is of limited importance in the maintenance of epizootic and epidemic sporotrichosis in Rio de Janeiro, whereas cats with confirmed clinical and mycological diagnosis are those with a greater chance of acting as sources of transmission since these animals often carry *Sporothrix* in skin lesions, nails and oral mucosa.

We believe that since Rio de Janeiro displays a world unique epidemic model of the disease, not fully understood, data on the infected and non-infected animals can be of major importance for future strategies of sporotrichosis prevention and control. Finally, considering the importance of the current concept of “one health”, the experience here observed can be helpful for distinct epizootias and/or zoonosis.
